# The complete chloroplast genome of *Codonopsis lanceolata* (Campanulaceae)

**DOI:** 10.1080/23802359.2018.1508382

**Published:** 2018-09-10

**Authors:** Junki Lee, So-Yeon Kim, Jong-Sung Lim, Hyang Sook Chun, Kisung Kwon, Youngho Koh, Tae Sun Kang, Jung-Hwa Kang, Eun-Jeong Kim, Gyoungju Nah

**Affiliations:** a Genome Analysis Center at National Instrumentation Center for Environmental Management, Seoul National University, Seoul, Republic of Korea;; b Department of Food Science and Technology, Chung-Ang University, Ahnsung, Republic of Korea;; c New Hazardous Substances Team, National Institute of Food and Drug Safety Evaluation, Ministry of Food and Drug Safety, Ohsong, Republic of Korea;; d Hantaek botanical garden, Yongin, Republic of Korea

**Keywords:** *Codonopsis lanceolata;* medicinal plant, complete chloroplast genome, next generation sequencing

## Abstract

The complete chloroplast genome sequence of *Codonopsis lanceolata* was determined by next generation sequencing. The total length of chloroplast genome of *C. lanceolata* was 169,447 bp long, including a large single-copy (LSC) region of 85,253 bp, a small single-copy (SSC) region of 8060 bp, and a pair of identical inverted repeat regions (IRs) of 38,067 bp. A total of 110 genes was annotated, resulting in 79 protein-coding genes, 27 tRNA genes, and 4 rRNA genes. The phylogenetic analysis of *C. lanceolata* with related chloroplast genome sequences in this study provided the taxonomical relationship of *C. lanceolata* in the genus *Campanula*.

The genus *Codonopsis* is composed of ∼42 herbaceous perennial plants belonging to the Campanulaceae family which are native to Central, East and South Asia (Hong et al. 2011; He et al. 2015). *Codonopsis lanceolate* is enriched with saponin and has been widely used as medicinal plants effective for bronchitis, cough, spasm, and inflammation (Lee 1985; Lee et al. 2002). To date, the barcode marker system has not been well established, in spite of the importance of molecular authentication system of medicinal plants, including *C. lanceolate.* We reported complete chloroplast genome sequence of *C. lanceolata* which will serve as a source of future barcode marker development, as well as assist further phylogenetic study of genus *Codonopsis*.

The leaves of *C. lanceolata* were supplied from Hantaek botanical garden (www.hantaek.co.kr) in Yongin-si, Korea (37° 05′ 40.4” N, 127° 24′ 23.7” E). A genomic library of *C. lanceolata* was constructed and sequenced using an Illumina HiSeq platform. We obtained a total of 1.6 Gb high-quality paired-end reads and assembled through CLC_assembler (ver. 4.010.83648, CLC QIAGEN) with manual correction by paired-end reads mapping by CLC_mapper (ver. 4.010.83648, CLC QIAGEN) (Kim et al. 2015). The genes of the complete chloroplast genome were predicted using Ge-Seq with manual curations of start and stop codons (Tillich et al. 2017).

The complete chloroplast genome of *C. lanceolata* (GenBank accession no. MH018574) has a circular molecular structure of 169,447 bp in length with 38.25% of GC content. Like typical chloroplast genomes, the complete chloroplast genome of *C. lanceolata* was comprised of four regions: a large single-copy (LSC) region of 85,253 bp, a small single-copy (SSC) region of 8060 bp, and a pair of inverted repeat (IRa and IRb) regions of 38,067 bp. We annotated 110 genes, including 79 protein-coding genes, 27 tRNA genes, and 4 rRNA genes. In addition to gene annotation, 26 simple sequence repeats were identified with the minimum repeat number of 21, 4, and 1 for mono-, di-, and tri- nucleotides, respectively, using MISA tool (http://webblast.ipk-gatersleben.de/misa/) (Beier et al. 2017).

We performed the phylogenetic analysis of *C. lanceolata* with 10 species of Campanulaceae family using MAFFT (ver. 7.271) by Neighbor-Joining tree analysis of MEGA 7.0 with 1000 bootstraps (Katoh et al. 2002; Kumar et al. 2016). The phylogenetic analysis revealed genus *Codonopsis* was closely related to genus *Campanula* (Figure 1).

**Figure 1.**
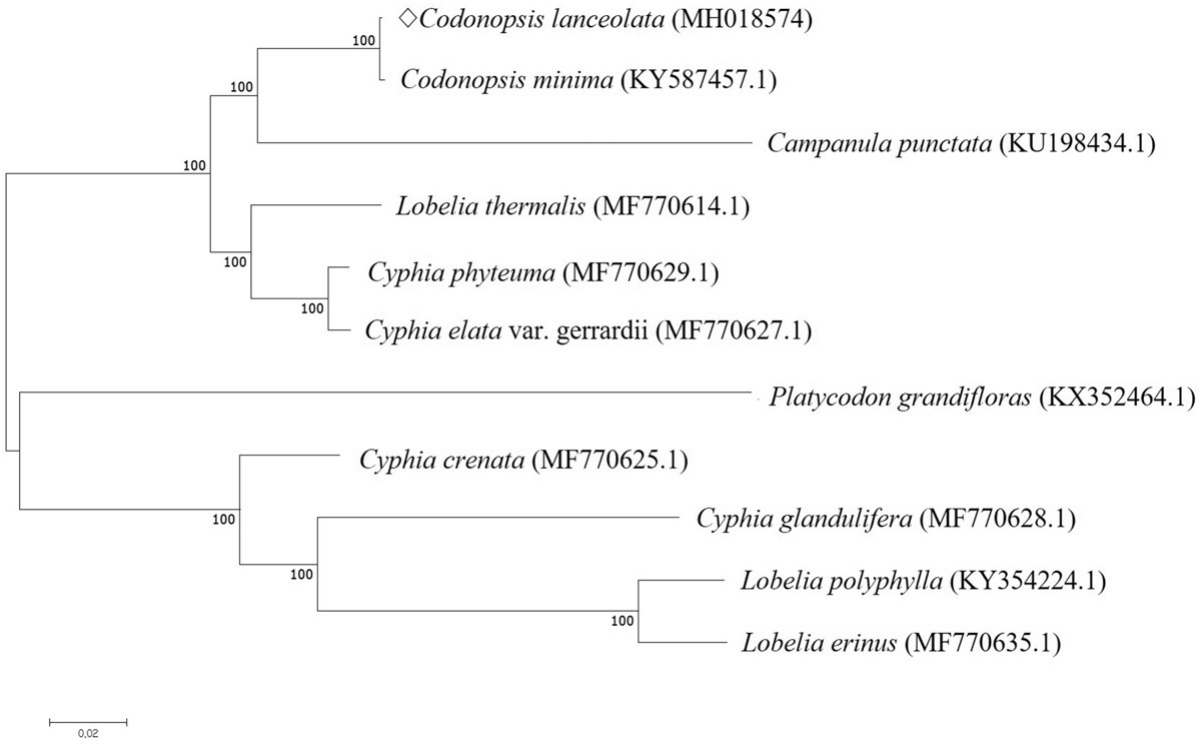
Phylogenetic analysis of *C. lanceolata* with 10 related species by Neighbor-Joining (NJ) methods. Numbers in the nodes are the bootstrap values from 1000 replicates.
